# Health Benefits of Urban Allotment Gardening: Improved Physical and Psychological Well-Being and Social Integration

**DOI:** 10.3390/ijerph14010071

**Published:** 2017-01-12

**Authors:** Masashi Soga, Daniel T. C. Cox, Yuichi Yamaura, Kevin J. Gaston, Kiyo Kurisu, Keisuke Hanaki

**Affiliations:** 1 Department of Urban Engineering, School of Engineering, The University of Tokyo, 7-3-1, Hongo, Bunkyo, Tokyo 113-8656, Japan; kiyo@env.t.u-tokyo.ac.jp (K.K.); hanaki@env.t.u-tokyo.ac.jp (K.H.); 2 Environment and Sustainability Institute, University of Exeter, Penryn, Cornwall TR10 9FE, UK; D.T.C.Cox@exeter.ac.uk (D.T.C.C.); K.J.Gaston@exeter.ac.uk (K.J.G.); 3 Forestry and Forest Product Research Institute, Matsunosato 1, Tsukuba 305-8687, Japan; yamaura@ffpri.affrc.go.jp

**Keywords:** agriculture, community health, ecosystem services, extinction of experience, green infrastructure, health promotion, nature experiences, urban greenspace, urbanisation, well-being

## Abstract

With an ever-increasing urban population, promoting public health and well-being in towns and cities is a major challenge. Previous research has suggested that participating in allotment gardening delivers a wide range of health benefits. However, evidence from quantitative analyses is still scarce. Here, we quantify the effects, if any, of participating in allotment gardening on physical, psychological and social health. A questionnaire survey of 332 people was performed in Tokyo, Japan. We compared five self-reported health outcomes between allotment gardeners and non-gardener controls: perceived general health, subjective health complaints, body mass index (BMI), mental health and social cohesion. Accounting for socio-demographic and lifestyle variables, regression models revealed that allotment gardeners, compared to non-gardeners, reported better perceived general health, subjective health complaints, mental health and social cohesion. BMI did not differ between gardeners and non-gardeners. Neither frequency nor duration of gardening significantly influenced reported health outcomes. Our results highlight that regular gardening on allotment sites is associated with improved physical, psychological and social health. With the recent escalation in the prevalence of chronic diseases, and associated healthcare costs, this study has a major implication for policy, as it suggests that urban allotments have great potential for preventative healthcare.

## 1. Introduction

More than three billion people currently live in urban areas and this figure is projected to soar to six billion (approximately 70% of the world’s population) within 30 years [1]. This increased trend towards urbanisation has profound implications for human health, with urban lifestyles being associated with a number of adverse health outcomes, such as decreased levels of physical activity [2], increased consumption of high-energy foods [3] and social and psychological stress [4,5]. Indeed, chronic and non-communicable diseases (e.g., depression, anxiety, high blood pressure, diabetes) are becoming increasingly common in urban areas [6,7,8]. In consequence, promoting healthy lifestyles, which is regarded as a key part of the preventative health care approach [9], in towns and cities is a major challenge in the 21st century [10].

Nature in cities has great potential to provide an inexpensive intervention to assist in addressing many of these issues [11,12]. It is widely recognised that regular contact with nature is related to a range of favourable health outcomes, such as increased psychological well-being [13,14], general health [15] and social cohesion [16]. Indeed, epidemiological studies show that the quantity of neighbourhood greenspace is significantly related to the incidence of various chronic and non-communicable diseases, including depression and anxiety symptoms [17], diabetes and obesity [18], and circulatory and heart disease [19]. It is therefore increasingly accepted that regular doses of nature are an essential part of maintaining a healthy lifestyle [11] and, in some instances, can be used as a preventative measure [14,20].

Allotment gardening provides city dwellers with opportunities to experience nature in their daily life. Research has shown that allotment gardens, and other types of community gardens, deliver various health benefits to people, including recovery from stress and fatigue [21,22,23], increased self-esteem [24], improved life satisfaction [25] and better social networks [26,27]. Recent studies also showed that engagement with allotment gardening is related to improved physical health [24,25]. Nevertheless, the majority of evidence of these beneficial effects has, to date, been based on qualitative and descriptive approaches [21,23,27], and empirical evidence from quantitative analysis is still scarce [24,25]. Moreover, as most of the existing published research has been performed in Western societies (Europe and North America), it is unclear whether and to what extent the findings can be generalised more widely [20].

Given this wide variety of associated health benefits, it is perhaps unsurprising that allotment gardening is today a popular pastime in many countries [28,29]. There are numerous allotments in Europe; in the U.K. alone, for example, there are estimated to be *c*. 330,000 allotment plots across the country [30]. In Asia, where urban design and culture is very different, allotment gardening appears to be similarly popular; indeed in Japan, there are currently estimated to be *c*. 190,000 allotment plots (note that more than 80% of this figure comes from urban areas), with both their number and total size increasing annually [31]. Given the scale of, and the rising interest towards, allotment gardening, there is great potential for its use to promote healthy lifestyles for urban communities.

As the world’s largest mega-city, Tokyo, Japan, provides an interesting opportunity to examine the health benefits of allotment gardening. It has an extremely high population density (>6000 people per km^2^; [32]), with more than 80% of people living in apartments with no access to private gardens [33]. This is coupled with very low per capita public green space (3 m^2^; [34]), resulting in people’s daily experiences of nature being extremely limited. Thus, publicly-owned allotments are likely to play a key role in allowing residents to actively engage with nature and, thus attain health benefits there from. Here, using the Tokyo population, we quantify the effects of allotment gardening on physical, psychological and social health. We surveyed 332 people to first test whether residents who used allotments (hereinafter “gardeners”) reported better health across five health outcomes, compared to those who did not (hereinafter “non-gardeners”). We then examined possible relationships between the frequency and duration of allotment gardening and health outcomes.

## 2. Materials

### 2.1. Site Description

**Figure 1:**
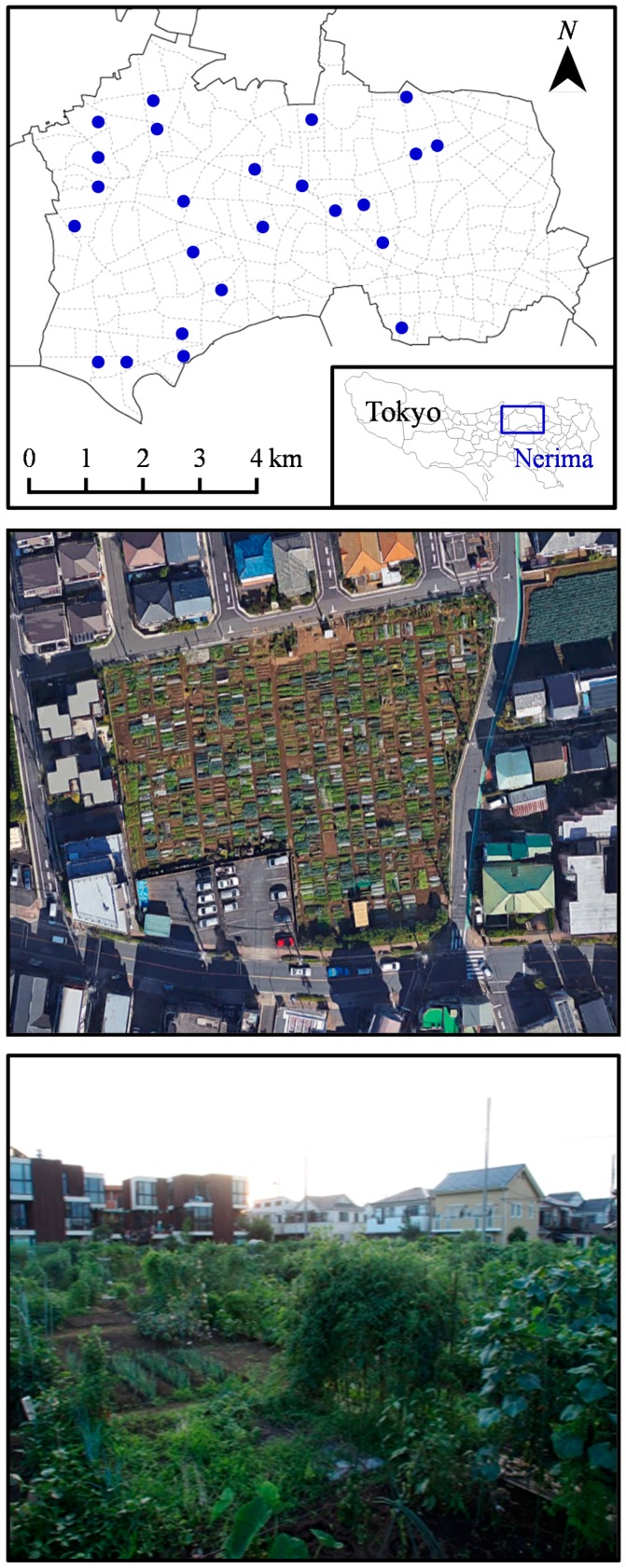
Map of Nerima, Tokyo, with 24 survey allotments (blue circles), and example pictures of an allotment garden. Broken lines on the top panel indicate administrative district borders.

This study was conducted in the city of Nerima, one of the 23 wards of the Tokyo metropolis (Figure 1). Nerima is located in the centre of Tokyo, covers 48.1 km^2^ and has a population of 722,492 residents (*c*. 15,000 people per km^2^) [35]. The socioeconomic composition of its residents is comparable to that of other regions of Tokyo [35]. In Nerima, there are currently 24 publicly-owned allotments (1836 plots), which are available to Nerima residents and predominantly located within the city (Figure 1). These allotment sites range between 0.05 and 0.47 ha.

### 2.2. Participants

The study was approved by the research ethics committee at the University of Tokyo (no. 27-276). All respondents provided written informed consent.

Allotment gardeners were recruited from all 24 allotments in the spring–summer seasons in 2016 (from the beginning of May to the end of June), a period when people are most likely to be engaged in allotment gardening. At each allotment, a four-page questionnaire, written in Japanese, was delivered face-to-face in situ (09:00 to 18:30) to as many gardeners as possible, who were asked to complete and return it to an envelope at the entrance. To prevent the duplication of sample data, we delivered only one questionnaire per plot. In total, 184 questionnaires were delivered.

Non-gardeners were recruited via a postal questionnaire survey of 1000 residents in Nerima. Respondents were selected at random and were dispersed geographically across the city. We posted a four-page questionnaire (with a return envelope) to each respondent’s mailbox in early June 2016. Similar to the survey of gardeners, only one questionnaire was delivered per household. In the questionnaire, we checked whether non-gardeners did not participate in allotment gardening. If a respondent noted that s/he participated in allotment gardening, they were excluded from the analysis (*n* = 2). After four weeks, the number of responses decreased sharply and the survey was terminated on 5 July 2016.

### 2.3. Health Outcomes

Respondents provided self-reported information on five health outcomes (see Supplementary Materials for full description):

(1) Perceived general health was measured by a single question “How do you rate your health in general?” Responses were scored on a five-point scale, ranging from 1 (Poor) to 5 (Excellent). This measure is known to be related to morbidity and mortality rates and is a strong predictor of health status [36,37]. In this section, gardeners were also asked to report how strongly they felt that their perceived general health was improved relative to before participating in gardening, which was scored on a four-point scale ranging from “No change” to “Strongly improved.”
(2) Subjective health complaints were measured with a 10-item symptom checklist (feeling fatigue or tired, poor appetite, difficulty falling asleep, headache, constipation, lack of facial expression, hypothermia, catching a cold easily, out of breath during daily physical activities, feeling muscle weakness), which was modified from the Subjective Health Complaints Inventory [38]. The total number of health complaints was used as a measure of subjective health complaints, ranging from 0 to 10.
(3) Body mass index (BMI) was calculated using self-reported height and weight. BMI is related to overall health and obesity, cardiovascular mortality and morbidity [39]. BMI values in excess of 25 and 30 are considered as overweight and obese, respectively.
(4) Mental health was assessed using the 12-item General Health Questionnaire, which is the most extensively used self-report instrument for measuring common mental disorders, such as anxiety and depression [40]. For each question, responses indicating distress score 1 and those indicating no or limited distress score 0. The scores across the 12 items were summed, ranging from 0 to 12.
(5) Social cohesion was assessed with the Social Cohesion and Trust Scale [41]. This scale asked respondents how they agreed with statements about their neighbours. Responses of each of these items were scored on a four-point scale ranging from 0 (Disagree strongly) to 4 (Agree strongly). The scores across the five items were summed, ranging from 0 to 20.

### 2.4. Socio-Demographic and Lifestyle Variables

We measured socio-demographic and lifestyle variables about respondents that are known to influence health (see Table S1 for details). Socio-demographic variables included gender, age, annual household income, and employment status. Lifestyle variables included the frequency of smoking, drinking alcohol and vegetable intake, and levels of physical activity (average number of days per week participating in at least 30 min of at least moderate physical activity). We also measured respondents’ emotional relatedness to nature, as this is known to influence psychological well-being [42]. We used the short version of the Nature Relatedness Scale [43], which ranged from 1.0 to 5.0.

### 2.5. Motivation, Frequency and Duration of Gardening

The questionnaire for gardeners included an additional section that aimed to investigate their motivation for, frequency and duration of allotment gardening. For the motivation for gardening, respondents were asked to select one or more items from the following list: physical exercise; getting in shape; having contact with nature; learning more about nature; relaxing; taking a mental break; enjoying social interaction; growing vegetables to eat; other. Frequency of gardening was measured by the average number of times respondents participated in allotment gardening per month over the spring–summer season. Average duration of allotment visits was measured as self-reported time (minutes) spent in an allotment per visit. We then estimated monthly total minutes by multiplying the frequency and average duration of allotment visits.

### 2.6. Statistical Analyses

All analyses were performed in the software R (ver. 3.2.3; [44]). We used chi-square tests and analysis of variance (ANOVA) to investigate the differences in socio-demographic and lifestyle variables between gardeners and non-gardeners. To examine the effects of participating in, and the frequency and duration of, allotment gardening on respondents’ health, we used linear regression for subjective health complaints, BMI, mental health, and social cohesion, and cumulative link models for perceived general health. We built a separate model for each health outcome, using the health outcome as the response variable, and respondent type (gardeners/non-gardeners), frequency and duration of gardening and nine socio-demographic and lifestyle variables as the explanatory variable. Following Shanahan et al. [14], we generated three predictor model sets for each of the five health outcomes: (i) all socio-demographic and lifestyle variables (but excluding respondent type and frequency and duration of gardening); (ii) socio-demographic and lifestyle variables plus respondent type; (iii) socio-demographic and lifestyle variables plus frequency and duration of gardening. The effect sizes of the categorical variables were relative to a base factor level as follows: “non-gardeners” (respondent types), “less than ¥3,000,000 (*c.* $30,000)” (household income), “female” (gender), and “regular employee” (employment status). The most parsimonious model was selected using Akaike’s Information Criterion (AIC). Following Richards [45], model averaging was performed, and we retained all models where ΔAIC < 6. By averaging over a subset of models, we calculated the mean estimates and 95% confidence intervals (CI) for each explanatory variable.

## 3. Results

### 3.1. Sample Description

A total of 332 valid questionnaires were returned and used in the analysis (165 gardeners and 167 non-gardeners). A response rate of 89.7% and 19.7% was obtained for gardeners and non-gardeners, respectively. The proportion of males and of retirees was higher in gardeners compared to non-gardeners, and the frequency of vegetable intake and drinking alcohol was higher in gardeners (Table 1 and Table 2). There was no significant difference between the groups regarding age, household income, nature relatedness, physical activity levels, and the frequency of smoking (Table 1 and Table 2).

**Figure 2:**
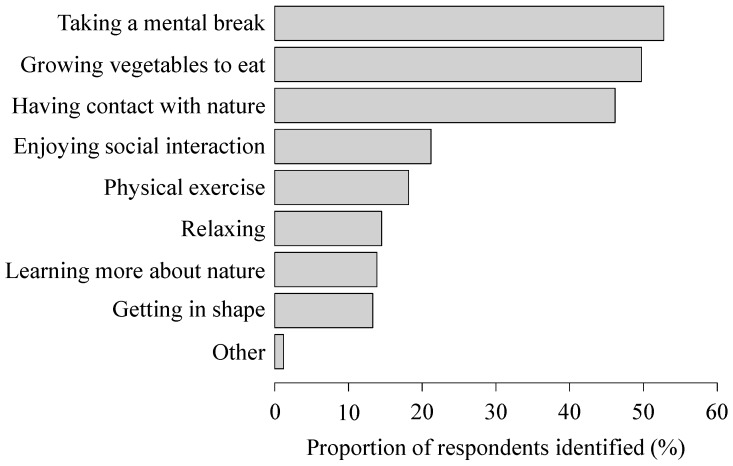
Gardener’s motivations for allotment gardening. Note that multiple answers were allowed.

Gardeners reported a variety of motivations for participating in allotment gardening, the main one being taking a mental break, followed by growing vegetables to eat, having contact with nature, and enjoying social interaction (Figure 2).

### 3.2. Comparison of Gardeners and Non-Gardeners

The estimates from model averaging showed that respondent type (i.e., gardeners) had significant positive effects on perceived general health (β = 1.40) and social cohesion (β = 0.57) and negative effects on subjective health complaints (β = −0.43) and general mental health (β = −0.91), respectively (Table 3). In other words, gardeners, compared to non-gardeners, reported significantly better perceived general health and mental health, lower numbers of subjective health complaints, and greater social cohesion. There was no significant relationship between respondent type and BMI (Table 3): BMI did not differ between gardeners and non-gardeners.

### 3.3. Frequency and Duration of Gardening

Of the 165 gardeners, the average number of visits to allotments was 15.7 (SD = 10.9) times per month, with the average duration of each visit being 80.0 (SD = 64.9) minutes. The estimated average duration of all allotment visits per month was 1257.9 (SD = 1541.8) minutes. Model averaging showed that neither the frequency nor duration had significant influences on five health outcomes (Table 3; Figure S1).

## 4. Discussion

In the present study, we have shown that allotment gardeners, compared to non-gardeners, reported significantly better perceived general health, mental health and social cohesion, and these findings hold even after controlling for the socio-demographic and lifestyle variables. Our study provides support for the notion that participating in allotment gardening is associated with improved health [20,46]. Below we discuss several, not mutually exclusive, potential mechanistic pathways through which allotment gardening could improve respondents’ health.

First, allotments, along with other types of urban greenspace, provide people with an opportunity to interact directly with nature. Indeed, exposure to nature benefits psychological health through mechanistic pathways that are now well established, namely attention restoration theory [47] and stress reduction theory [48]. Notably, in the present study, approximately half of gardeners identified having contact with nature as a motivation for allotment gardening. This result indicates that, even in a highly urbanised area like Tokyo, where natural environments and features have largely been lost, people still seek meaningful regular contact with nature and actually recognise, and appreciate, some of the beneficial effects from nature experiences [49]. Given this, urban allotments have the potential to play an important role in not only helping people to maintain their psychological health, but also countering the ongoing loss of human–nature interactions—the “extinction of experience” [49,50].

Second, allotment gardening involves a moderate intensity of physical activity, which promotes people’s physical fitness and health, as well as delivering additional psychological health benefits. We observed that gardeners spent more than 20 h per month, on average, engaging with gardening activities. Since regular physical exercise promotes general health conditions and reduces the risk of a range of chronic diseases [51], this result has a significant implication for public health. Indeed, gardeners reported a significantly lower number of health complaints compared to non-gardeners. However, we did not find clear differences in BMI, a reliable indicator of overweight or obesity, between gardeners and non-gardeners, which is not congruent with the findings of recent studies performed in Europe [24,25]. One possible reason for this is that being overweight or obese is not a severe health problem in our study region: Japan has one of the lowest rates of obesity across the world [52]. The extent of health benefits people obtain from allotment gardening is likely to depend on the social and cultural contexts of the society.

Third, allotment gardening provides people with opportunities to interact with others in a social setting (e.g., transmitting knowledge of vegetable cultivation and bartering vegetables), thus promoting a sense of community and social ties. Indeed, we observed significantly higher levels of social cohesion in gardeners compared to non-gardeners. This result strengthens the notion that allotment and community gardens within urban areas generate social capital through the development of a cohesive social network [27]. Increased social cohesion offers important implications for public health, as it is known to be a key determinant of psychological health [53].

Lastly, participating in allotment gardening is likely to increase people’s vegetable consumption. We observed that gardeners reported significantly higher frequency of vegetable intake (Table 1), which would undoubtedly be due to increased opportunities to consume vegetables grown in their own allotment plots. Indeed, in our study, growing vegetables to eat was the second-most important motivation for gardening. More importantly, participating in allotment gardening is also likely to improve people’s knowledge of nutrition and vegetable preferences, which may, in turn, increase their vegetable consumption [54]. Because greater vegetable intake is associated with lower risk of major chronic diseases [55], such a behavioural change can, from a long-term perspective, have a huge impact on public health outcomes.

This study used a cross-sectional design, which inevitably has both advantages and limitations. The main advantage is that it allows comparison of the effects of many different variables (risk factors) simultaneously. Indeed, using regression analysis and model averaging approaches, we showed that participating in allotment gardening had a significant impact on respondents’ health as well as other socio-demographic and lifestyle variables. The limitation, on the other hand, is that this study design cannot definitively establish cause–effect relationships. Other studies have, however, provided such support (see [20]). Van den Berg and Custer [56], for example, investigated people’s psychological health before and after 30 min of outdoor gardening and found a significant reduction in stress levels after the treatment. More importantly, their study suggested that neuroendocrine processes mediate the relationship between gardening and improved psychological health. Clinical studies employing horticultural therapy have also observed that gardening activities ameliorate the severity of depression and anxiety symptoms in patients with psychological disorders and its effect persists over a few months (e.g., [57]). Further studies tracking people’s health over time would provide valuable information for understanding the long-term effects of allotment gardening.

Neither frequency nor duration of gardening significantly affected respondents’ health (Figure S1). In other words, gardeners who used an allotment at a low frequency, and for a short duration, reported similar levels of health compared to those who did so regularly and for longer. This is a critically important finding because it indicates that even a low frequency, and short duration, of gardening is associated with improved health outcomes. Indeed, a recent UK study suggested that a single session of 30 min of allotment gardening produces a significant psychological health benefit to people [24]. Although more evidence is necessary, our study implies that allotment gardening is an effective health promotion tool, which can be easily fitted into urban lifestyles.

We found no difference in Nature Relatedness scores (an indicator of an individual’s emotional bonds with nature) between gardeners and non-gardeners. This result is notable because it suggests that the health benefits of allotment gardening are not limited to specific groups of people (i.e., those who feel strong affinities to nature), rather they are applicable to the wider population. Also, there was no difference in socioeconomic (household income) levels between gardeners and non-gardeners. Furthermore, a large proportion of the gardener sample was comprised of older people—those who may be more likely to be physically inactive and socially isolated. These findings highlight that urban allotments can contribute to reducing health inequality within populations through the provision of equal opportunities to interact with nature [58].

We acknowledge that there are some limitations to our study. First, as this study employed a non-randomised setting in the study design, one must be careful with drawing definite conclusions. Second, although we considered various socio-demographic and lifestyle variables in the analysis, there may be other factors affecting people’s health outcomes. Third, we used a self-reported questionnaire to determine the relationship between participating in allotment gardening and health. A strength of self-reported health questionnaires is the ability to reach respondents who might not be willing to participant in the more comprehensive health study; however, there is some limitation in that self-reported health responses do not always accurately reflect actual nature exposure or health.

## 5. Conclusions

Globally, the prevalence of chronic and non-communicable diseases is increasing at an alarming rate. In Japan alone, more than 10 million people are identified as having hypertension and 9 million with diabetes, the treatment of which has been estimated to have an annual cost to the national healthcare budget of over 10 billion dollars [59]. Mental health issues are also on the rise in Japan, with over one million people estimated to be suffering from depression [60]. Given these conditions, our results have potentially major implications for healthcare policy, as they suggest that urban allotment gardening has potential for improving healthy lifestyles and helping to prevent or ameliorate risk factors to health. Policy-makers and health practitioners should view allotment gardening as an important health promotion tool and encourage people to participate in it. Indeed, since many allotments require relatively small pieces of land, creating new or expanding existing allotments as a “land-sharing” urban form [61,62] may be relatively straightforward to achieve even in a densely-populated area like Tokyo. Furthermore, given the associated benefits to the provision of multiple ecosystem services, such as ameliorating the heat island effect and improving carbon storage, and the habitat for local biodiversity [63], creating and maintaining urban allotments would bring wide-ranging environmental benefits to the society where they would be felt most.

## Figures and Tables

**Table 1:** Individual characteristics (gender, income, employment status, and the frequency of smoking, drinking alcohol, and vegetable intake) of the study sample. Statistical differences were tested with a chi-squared test.

Characteristics		Gardeners		Non-Gardeners		Statistical Significance
		*N*	%	*N*	%	
Gender	Female	52	31.9	92	58.2	*χ*^2^ = 21.43, *df* = 1, *p* < 0.001
	Male	111	68.1	66	41.8	
Household income	Less than ¥3,000,000 ($30,000)	40	37.4	60	40.5	*χ*^2^ = 3.58, *df* = 5, *p* = 0.61
	¥3,010,000–5,000,000	25	23.4	38	25.7	
	¥5,010,000–7,000,000	13	12.1	22	14.9	
	¥7,010,000–10,000,000	17	15.9	13	8.8	
	¥10,010,000–15,000,000	9	8.4	10	6.8	
	Over ¥15,000,000	3	2.8	5	3.4	
Employment status	Student	3	1.9	5	3.2	*χ*^2^ = 14.52, *df* = 7, *p* = 0.04
	Housewife/househusband	30	19.2	37	23.7	
	Irregular employee	11	7.1	21	13.5	
	Self-employed	13	8.3	12	7.7	
	Regular employee	42	26.9	28	17.9	
	Unemployed	13	8.3	20	12.8	
	Retiree	43	27.6	28	17.9	
	Others	1	0.6	5	3.2	
Smoking	Never	140	87.5	138	87.3	*χ*^2^ = 0.90, *df* = 3, *p* = 0.82
	Seldom	3	1.9	2	1.3	
	Sometimes	15	9.4	14	8.9	
	Often	2	1.3	4	2.5	
Drinking alcohol	Never	49	30.6	59	37.3	*χ*^2^ = 8.57, *df* = 3, *p* = 0.04
	Seldom	39	24.4	47	29.7	
	Sometimes	50	31.3	44	27.8	
	Often	22	13.8	8	5.1	
Vegetable intake	Seldom	3	1.9	17	10.7	*χ*^2^ = 35.22, *df* = 2, *p* < 0.001
	Sometimes	71	44.1	104	65.4	
	Often	87	54.0	38	23.9	

**Table 2:** Individual characteristics (age, nature relatedness, and physical activity levels) of the study sample. Statistical differences were tested with analysis of variance (ANOVA).

Characteristics	Gardeners		Non-Gardeners		Statistical Significance
	Mean	SD	Mean	SD	
Age (years)	61.9	17.1	61.0	16.4	F (1302) = 0.25, *p* = 0.62
Nature relatedness	3.6	0.6	3.6	0.6	F (1319) = 0.31, *p* = 0.58
Physical activity levels (days per week)	3.9	2.3	3.9	3.3	F (1306) = 0.001, *p* = 0.98

**Table 3:** The relationship between five health outcomes (the response variables), socio-demographic and lifestyle variables, and participation in, and frequency and duration of, allotment gardening.

Explanatory Variables	Perceived General Health	Subjective Health Complaints	BMI	General Mental Health	Social Cohesion
Model (i)					
Age	**−0.02 (0.01)** **	−0.01 (0.01)	0.02 (0.01)	−0.02 (0.01)	**0.04 (0.02)** *
Gender (male)	**0.55 (0.27)** *	**−0.46 (0.19)** *	**2.40 (0.38)** ***	−0.28 (0.45)	0.72 (0.61)
Nature relatedness	**0.50 (0.23)** *	0.08 (0.17)	**−0.76 (0.33)** *	0.26 (0.35)	0.43 (0.49)
Household income (¥3,010,000–5,000,000)	NA	−0.33 (0.24)	NA	−0.52 (0.51)	0.28 (0.71)
Household income (¥5,010,000–7,000,000)	NA	−0.50 (0.31)	NA	−0.82 (0.65)	0.56 (0.91)
Household income (¥7,010,000–10,000,000)	NA	−0.08 (0.34)	NA	−1.26 (0.71)	0.12 (1.00)
Household income (¥10,010,000–15,000,000)	NA	−0.47 (0.45)	NA	−1.65 (0.95)	2.4 (1.32)
Household income (over ¥15,000,000)	NA	0.13 (0.54)	NA	−1.84 (1.12)	1.76 (1.57)
Employment status (student)	3.32 (1.38)	NA	NA	−3.42 (1.84)	NA
Employment status (housewife/househusband)	0.06 (0.51)	NA	NA	0.73 (0.70)	NA
Employment status (irregular employee)	0.04 (0.53)	NA	NA	0.25 (0.78)	NA
Employment status (self-employed)	0.31 (0.51)	NA	NA	−0.71 (0.78)	NA
Employment status (unemployed)	−0.48 (0.56)	NA	NA	0.74 (0.86)	NA
Employment status (retiree)	−0.46 (0.44)	NA	NA	0.03 (0.73)	NA
Employment status (others)	0.17 (0.94)	NA	NA	−1.45 (1.40)	NA
Frequency of smoking	−0.32 (0.18)	0.16 (0.13)	0.00 (0.26)	**0.56 (0.27)** *	0.65 (0.38)
Frequency of drinking alcohol	0.04 (0.14)	0.04 (0.10)	0.10 (0.20)	**0.45 (0.21)** *	0.54 (0.29)
Frequency of vegetable intake	**0.81 (0.22)** ***	**−0.41 (0.16)** **	0.12 (0.32)	**−1.24 (0.34)** ***	0.51 (0.47)
Physical activity levels	**0.15 (0.06)** **	**−0.06 (0.03)** *	0.00 (0.07)	−0.06 (0.07)	0.01 (0.10)
Model (ii)					
Model (i) + Respondent type (gardener)	**1.40 (0.29)** ***	**−0.43 (0.21)** *	0.56 (0.39)	**−0.91 (0.42)** *	**1.57 (0.57)** ***
Model (iii)					
Model (i) + Frequency of gardening (times per month)	0.01 (0.02)	−0.01 (0.01)	−0.02 (0.03)	−0.01 (0.03)	0.08 (0.04)
Model (i) + Duration of gardening (monthly total minutes)	0.06 (0.55)	−0.62 (0.32)	−0.04 (0.87)	−0.48 (0.64)	0.96 (1.14)

Three models for each response variable are shown: (i) socio-demographic and lifestyle variables only; (ii) socio-demographic and lifestyle variables plus respondent type (gardener or non-gardener); (iii) socio-demographic and lifestyle variables plus frequency and duration of gardening. Model averaged coefficients are shown with standard error in brackets. Bold text indicates significant results (* *p* < 0.05, ** *p* < 0.01, *** *p* < 0.001). Positive coefficients indicate that scores for each health outcome were higher with higher values of the explanatory variables (note that high scores indicate worse health for subjective health complaints, body mass index (BMI) and general mental health). NA: Variables that were not retained in the top models where ΔAIC <6 (see also the main text).

## Supplementary Materials

The following are available online at www.mdpi.com/1660-4601/14/1/71/s1, Figure S1: Relationships between the frequency and duration of allotment gardening and five health outcomes, Table S1: Nine socio-demographic and lifestyle variables measured in this study.
