# Supplementary material for: Health Benefits of Urban Allotment Gardening: Improved Physical and Psychological Well-Being and Social Integration
Source: Int J Environ Res Public Health. 2017 Jan 12;14(1):71. doi: 10.3390/ijerph14010071 (PMC5295322; doi:10.3390/ijerph14010071)
Supplement: Supplementary file 1 [file ijerph-14-00071-s001.pdf]

Supplementary Materials: Health Benefits of Urban Allotment Gardening: Improved Physical and Psychological Well-Being and Social Integration

Masashi Soga, Daniel T. C. Cox, Yuichi Yamaura, Kevin J. Gaston, Kiyo Kurisu and Keisuke Hanaki

1. Methodology

1.1. Measurement of Five Health Outcomes

Perceived general health was measured by a single question "How do you rate your health in general?" which was adapted from the SF-36 Health Survey. Responses were scored on a five-point scale, ranging from 1 (Poor) to 5 (Excellent). This measure is known to be related to morbidity and mortality rates and is a strong predictor of health status [1,2].

Subjective health complaints were measured with a 10-item symptom checklist, which was modified from the Subjective Health Complaints Inventory [3]. Respondents were asked a question "Have you experienced the following ten health complaints in the last few weeks: feeling fatigue or tired, poor appetite, difficulty falling asleep, headache, constipation, lack of facial expression, hypothermia, catching a cold easily, out of breath during daily physical activities, feeling muscle weakness?" They were permitted to choose more than one health complaint. The total number of health complaints was used as a measure of subjective health complaints, ranging from 0 to 10 (high scores indicate worse health).

Body mass index (BMI) was calculated by using self-reported height and weight. BMI is considered an indicator of overall health, as has been shown to be a valid measure of obesity, cardiovascular mortality and morbidity [4]. BMI values in excess of 25 and 30 are considered as overweight and obese, respectively.

Mental health was assessed by using the 12-item General Health Questionnaire (GHQ-12), which is the most extensively used self-report instrument for measuring common mental disorders, such as anxiety and depression [5]. Respondents were asked to report how their health states have been in general, over the past few weeks compared to usual. GHQ-12 includes six positive (e.g., "Feeling reasonably happy") and six negative mood states (e.g., "Feeling unhappy and depressed"), and four levels of responses were given (Not at all; No more than usual; Rather more than usual; Much more than usual"). Responses indicating distress score 1 and those indicating no or limited distress score 0. The summed scores were used as a measure of mental health, ranging from 0 to 12 (high scores indicate worse health).

Social cohesion was assessed with the revised version of the Social Cohesion and Trust Scale [6]. This scale included the following five statements: "People in this community are willing to help their neighbours"; "This is a close-knit community"; "People in this community can be trusted"; "People in this community generally do not get along with each other"; and "People in this community do not share the same values" (the last two items were reversed). Respondents were asked to report how strongly they agreed with each question (Do not know; Disagree strongly; Disagree; Agree; Agree strongly). Following the methodology by Shanahan et al. [7], responses were scored from 0 to 4, with "Do not know" scoring zero. The summed scores were used as a measure of social cohesion, ranging from 0 to 20.

1.2. Socio-Demographic and Lifestyle Variables

We measured nine respondents' socio-demographic and lifestyle variables as follow (Table S1).

Table S1. Nine socio-demographic and lifestyle variables measured in this study.

Variables	Description
**Socio-Demographic Variables**	
Gender	Measured as male or female.
Age	Measured as actual age at last birthday.
Nature relatedness	Respondents were asked to complete the short version of the Nature Relatedness Scale, a 6-item scale [43]. Items (e.g., "My ideal vacation spot would be a remote, wilderness area") were rated on a 5-point scale, from 1 (Disagree strongly) to 5 (Agree strongly). A total Nature Relatedness scale score was calculated by summing the individual scores and dividing by 6 (scores ranged from 1.0 to 5.0).
Household income (annual)	Measured on a 6-point scale: 1 = less than ¥3,010,000 (*c.* $30,000); 2 = ¥3,010,000–5,000,000 (*c.* $30,100–$50,000); 3 = ¥5,010,000–7,000,000 (*c.* $50,100–$70,000); 4 = ¥7,010,000–10,000,000 (*c.* $70,100–$100,000); 5 = ¥10,010,000–15,000,000 (*c.* $100,100–$150,000); 6 = over ¥15,000,000 (*c.* $150,000).
Employment status	Respondents were asked to select one of the following items: student; housewife/househusband; regular employee; irregular employee; self employed; unemployed; retiree; other.
**Lifestyle variables**	
Frequency of smoking	Measured on a 4-point scale: 1 = Never, 2 = Seldom, 3 = Sometimes, 4 = Often.
Frequency of drinking alcohol	Measured on a 4-point scale: 1 = Never, 2 = Seldom, 3 = Sometimes, 4 = Often.
Frequency of vegetable intake	Measured on a 3-point scale: 1 = Seldom, 2 = Sometimes, 3 = Often.
Physical activity levels	Measured as the average number of days per week on which they participate in at least 30 min of moderate level physical activity, such as cycling, walking or occupational activities.

2. Figure S1

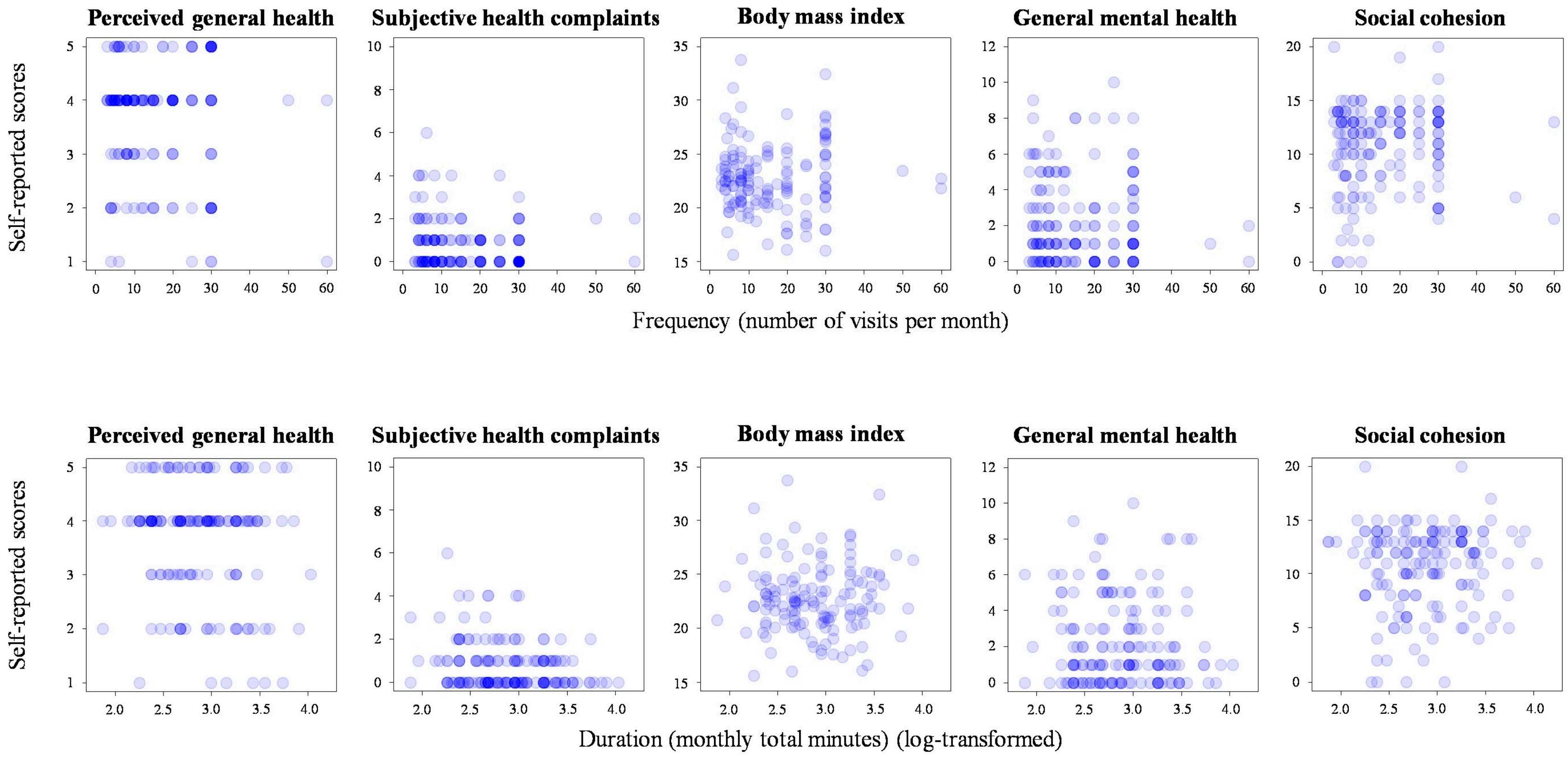

Figure S1. Relationships between the frequency and duration of allotment gardening and five health outcomes.

References

1. Idler, E.L.; Benyamini, Y. Self-rated health and mortality: A review of twenty-seven community studies. *J. Health Soc. Behav.* **1997**, *38*, 21–37.
2. Mossey, J.M.; Shapiro, E. Self-rated health: A predictor of mortality among the elderly. *Am. J. Public Health* **1982**, *72*, 800–808.
3. Eriksen, H.R.; Ihlebaek, C.; Ursin, H. A scoring system for subjective health complaints (SHC). *Scand. J. Public Health* **1999**, *27*, 63–72.
4. Gunnell, D.J.; Frankel, S.J.; Nanchahal, K.; Peters, T.J.; Smith, G.D. Childhood obesity and adult cardiovascular mortality: A 57-y follow-up study based on the Boyd Orr cohort. *Am. J. Clin. Nutr.***1998**, *67*, 1111–1118.
5. Goldberg, D. *General Health Questionnaire (GHQ-12)*; Nfer-Nelson: Windsor, UK, 1992.
6. Sampson, R.J.; Raudenbush, S.W.; Earls, F. Neighborhoods and violent crime: A multilevel study of collective efficacy. *Science* **1997**, *277*, 918–924.
7. Shanahan, D.F.; Bush, R.; Gaston, K.J.; Lin, B.B.; Dean, J.; Barber, E.; Fuller, R.A. Health benefits from nature experiences depend on dose. *Sci. Rep.* **2016**, *6*, 28551.

© 2017 by the authors; licensee MDPI, Basel, Switzerland. This article is an open access article distributed under the terms and conditions of the Creative Commons Attribution (CC-BY) license (http://creativecommons.org/licenses/by/4.0/).
